# Comparison of outcome prediction models post-stroke for a population-based registry with clinical variables collected at admission *vs*. discharge

**Published:** 2021-01-15

**Authors:** Kai-Cheng Hsu, Ching-Heng Lin, Kory R. Johnson, Yang C. Fann, Chung Y. Hsu, Chon-Haw Tsai, Po-Lin Chen, Wei-Lun Chang, Po-Yen Yeh, Cheng-Yu Wei

**Affiliations:** 1 School of Medicine, College of Medicine, China Medical University, Taichung, Taiwan; 2 Artificial Intelligence Center for Medical Diagnosis, China Medical University Hospital, Taichung, Taiwan; 3 Department of Neurology, China Medical University Hospital, Taichung, Taiwan; 4 Center for Artificial Intelligence in Medicine, Chang Gung Memorial Hospital, Taoyuan, Taiwan; 5 Bioinformatics Section, National Institute of Neurological Disorder and Stroke, National Institutes of Health, Bethesda, Maryland, USA; 6 Graduate Institute of Biomedical Sciences, China Medical University, Taichung, Taiwan; 7 Neurological Institute, Taichung Veterans General Hospital, Taichung, Taiwan; 8 Department of Neurology, Show Chwan Memorial Hospital, Changhua County, Taiwan; 9 Department of Neurology, St. Martin De Porres Hospital, Chiayi, Taiwan; 10 Department of Neurology, Chang Bing Show Chwan Memorial Hospital, Changhua County, Taiwan

**Keywords:** Stroke outcome, logistic regression, National Institutes of Health Stroke Scale, modified Rankin Scale, population-based stroke registry

## Abstract

**Aim:**

The ability to predict outcomes can help clinicians to better triage and treat stroke patients. We aimed to build prediction models using clinical data at admission and discharge to assess predictors highly relevant to stroke outcomes.

**Methods:**

A total of 37,094 patients from the Taiwan Stroke Registry (TSR) were enrolled to ascertain clinical variables and predict their mRS outcomes at 90 days. The performances (i.e., the area under the curves (AUCs)) of these independent predictors identified by logistic regression (LR) based on clinical variables were compared.

**Results:**

Several outcome prediction models based on different patient subgroups were evaluated, and their AUCs based on all clinical variables at admission and discharge were 0.85–0.88 and 0.92–0.96, respectively. After feature selections, the input features decreased from 140 to 2–18 (including age of onset and NIHSS at admission) and from 262 to 2–8 (including NIHSS at discharge and mRS at discharge) at admission and discharge, respectively. With only a few selected key clinical features, our models can provide better performance than those previously reported in the literature.

**Conclusion:**

This study proposed high performance prognostics outcome prediction models derived from a population-based nationwide stroke registry even with reduced LR-selected clinical features. These key clinical features can help physicians to better focus on stroke patients to triage for best outcome in acute settings.

## INTRODUCTION

Stroke is the second leading cause of death worldwide, affecting one in six adults, with an estimated 16.9 million cases of stroke in 2010^[1]^. Despite a 42% decrease in the number of strokes in high-income countries over the past four decades, stroke incidence in low- and middle-income countries has more than doubled^[1,2]^. Moreover, stroke for people living in low- and middle-income countries occurs 15 years earlier on average than those living in high-income countries^[1–3]^. Given this disparity, continued effort to improve stroke management remains a major health and socioeconomic challenge and priority worldwide.

Prediction of clinical outcome after stroke has been proposed and studied as one potential approach to improve stroke care management^[4]^. Specifically, the prediction of disability due to stroke can beneficially assist clinicians in making decisions regarding what tests to order, choice of therapy, how to communicate with the patient and family, as well as assist in reaching shared decisions^[5,6]^. Modeling for such prediction has been performed using different statistical techniques in conjunction with varying input information, and the success of these models has been varied and cross-evaluated^[7,8]^. What has been learned is that some modeling techniques perform better than others and that the input information with questionable quality selected to be included in modeling can influence prediction success, while the sample size of the information can generate bias and limit model generalizability. As such, further work in this regard is needed using high-quality input information with ample sample sizes to bring confidence to the prediction models as having high predictive power for disability post-stroke to be used in real-world medical practices.

This study aimed to identify prediction models for functional outcomes following stroke, to appraise these models using current guidelines, and to determine the pooled accuracy of identified models using a well-established national registry. The Taiwan Stroke Registry (TSR) is a national research database collecting data from over 64 hospitals and medical centers across the nation with stroke patients occurring over a 12-year period^[9,10]^. Using this database, we sought to develop a multiparametric tool to estimate the probability of achieving functional improvement and identify the important predictors at different key time points for the stroke outcome prediction, aiming to help clinicians triage the stroke patients for best outcomes.

## METHODS

### Patient data

Patients used in this study were from a nationwide prospective registry, the Taiwan Stroke Registry (TSR)^[9]^, collected from 64 participating stroke centers with a confirmed diagnosis of acute cerebrovascular disease^[10]^, i.e., ischemic and hemorrhagic stroke excluding transient ischemic attack and subarachnoid hemorrhage, with follow-ups up to 1 year. These patients went through clinical examinations, including computed tomography (CT) and/or magnetic resonance imaging (MRI) for the indexed event, following the international clinical guidelines for stroke. The demographic data, stroke type, National Institutes of Health Stroke Scale (NIHSS) scores, Barthel Index, blood pressure upon admission, medical history, pre-existing comorbidities, and treatment data as well as modified Rankin scale (mRS), medications and some discharge and follow-up data were recorded. Using TSR data as a human study protocol was approved by the institutional review board of all participating hospitals. The details of diagnosis, inclusion criteria, and collection of clinical variables of this registry have been presented elsewhere^[10]^. The full list of Taiwan Stroke Registry participating investigators is listed in Supplemental Appendix I.

### Data preprocessing

The TSR included the following four categories of datasets derived at different time points from admission to discharge and follow-ups: (1) demographic data; (2) measurement/diagnosis; (3) inpatient treatments and medications; and (4) discharge information plus follow-ups for up to one year. To ensure data quality, we performed data cleaning, validation, and resampling to remove missing data, outliers, and miss-coded data, as well as inconsistent clinical measurements before building the models^[11]^.

The primary goal of this study was to develop a multiparametric tool to estimate the probability of predicting the best functional improvement after stroke. The mRS is a clinician-reported and -quantified measure of disability and has been widely used to evaluate stroke outcomes^[12–14]^. Several studies attested to the validity and reliability of the mRS at different time points^[15–17]^, and we followed the model of functional mRS outcomes measured at 90 days divided into good outcome (mRS 0–2) and poor outcome^[18–20]^ (mRS 3–6) to determine what clinical variables and treatments showed significant predictive value for future disability status in studied stroke patients, and how accurate we can predict disability with this set of stroke big data-selected information.

### Statistical models

We used the independent *t*-test and chi-square test to compare the clinical variables between patient groups and utilized univariate logistic regression to calculate the univariate odds ratio of variables. Also, multivariable logistic regression (LR) was employed for feature selection and 90-day mRS (mRS_3m) outcome prediction. We built two supervised learning models to compare the prediction performances of functional assessments and clinical data in the registry. In the first model, all the clinical data in TSR at admission and discharge were included in LR. In the second model, the variables selected 100/100 times were then used as the input features to predict mRS_3m. Furthermore, we evaluated the two models in four different subgroups of patients, including male, female, ischemia, and hemorrhage for a better understanding of how these subgroups may affect the performance and prediction models in different populations of stroke patients. The flowchart of model construction with different subgroup datasets is shown in Supplemental Figure S1.

There were two clinical time points selected for our prediction models, i.e., admission and discharge. In models evaluated at admission, a total of 140 variables of information registered during admission were used. By adding to the data registered during admission, such as treatments, medications, and complications, a total of 262 variables were used in models at discharge. The 10-fold cross-validation method with 70% of the data for training and 30% for testing was used to select variables using stepwise Akaike information criterion (AIC)^[21]^. Variables were then included in the final models based on the criteria that they were selected ten times in each of the ten rounds of the bootstrap. The counts of selection and the coefficient of each selected variable were recorded and evaluated. Accuracy and area under the curve (AUC) were assessed by comparing model predictions to the actual mRS of patients in holdout test sets [Supplemental Figure S1]. The performances of each model were then evaluated and compared by statistical analysis using SPSS Statistics version 22 and RStudio version 1.2.1335 software.

## RESULTS

After data preprocessing and cross-validation steps, the final dataset used in this study contained 37,094 cases (mean age = 66.8, SD = 13.3, 60% male). We compared their clinical information, including demographic data, medical history, hospital areas, hospital rating scales, functional assessments, laboratory data, and treatment, between the good and poor outcome patient groups as shown in Table 1. The good outcome patients were found to be younger (age = 63.9 ± 12.8) and male-predominant (65%) with higher BMI (24.9). The average hospitalization days were 6.2 and 10.3 in the good and poor outcome groups, respectively. The TSR included ischemic and hemorrhagic patients, and there were more ischemic patients (92%) in the good outcome group. Patients with underlying diseases except dyslipidemia were prone to have poor outcomes. However, smoking and drinking were not correlated to poor outcomes. Patients from hospitals in the middle of Taiwan and regional hospitals tended to be in the poor outcome group. Poor functional status at admission and discharge led to poor outcomes, and higher hemoglobin and albumin were found to be correlated to good outcomes. Aspirin was found to be related to good outcomes; however, heparin, intra-arterial (IA) thrombolysis, intravenous tissue plasminogen activator (IV t-PA), Foley, and rehabilitation were related to poor outcomes.

We further evaluated the differences in NIHSS and mRS between admission, discharge, and functional outcomes at three months in the population [Supplemental Table S1]. About 28.74% of patients in this study showed no change in NIHSS between admission and discharge. Nearly one-fifth (17.93%) of patients showed significant improvement, which was defined by reduced NIHSS by 4 points (NIHSS_diff ≥ 4) at discharge as compared to that at admission^[22]^. The NIHSS_diff between −1 and −3 (i.e., moderate recovery) was found in 37.50% of all patients, and above 1 (i.e., deteriorated outcome) was found in 15.84% of all patients. In addition, 57.45% of patients showed no change in mRS between discharge and three months post-stroke (i.e., mRS_diff), and 36.25% of patients showed improvement (mRS_diff value of −1 to −5) during this period. Overall, more than 50% of patients improved functionally during hospitalization and became stationary between discharge and three months post stroke.

**Figure 1.**
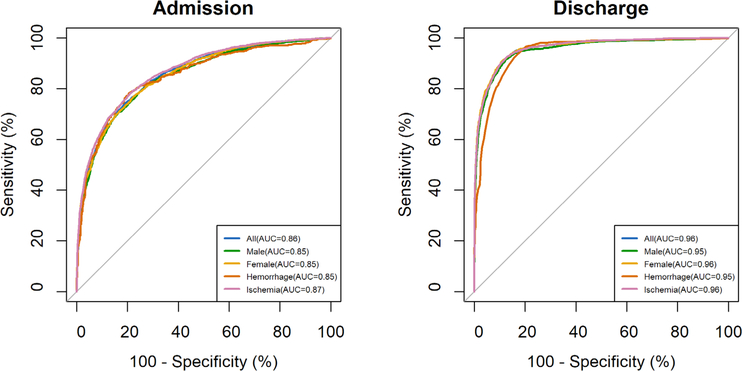
The ROC curves of admission and discharge models. The AUCs obtained at discharge were higher than those obtained at admission. AUCs: area under the curves

With a solid understanding of the population represented in the dataset, different prediction models were assessed and compared. The performances of stroke outcome prediction models using clinical data in different subgroups of patients at admission and discharge are listed in Table 2. By using all clinical data collected at admission (i.e., 140 variables), the best accuracy was 0.82 with AUC of 0.88. After feature selection by the LR method, 2 to 18 clinical data were selected in each subgroup as predictive input features to achieve similar performance obtained using all clinical variables. By using all clinical data available at discharge (i.e., 262 variables), an increase in accuracy and AUC was achieved compared to the performance obtained at admission. The best accuracy increased from 0.82 to 0.90, and the best AUC increased from 0.88 to 0.96. After feature selection again by the LR method, only 2 to 8 features in each subgroup were selected that could be used to achieve similar performance at discharge. The receiver operating characteristic curves of prediction models obtained at admission and discharged are shown in Figure 1.

**Figure 2.**
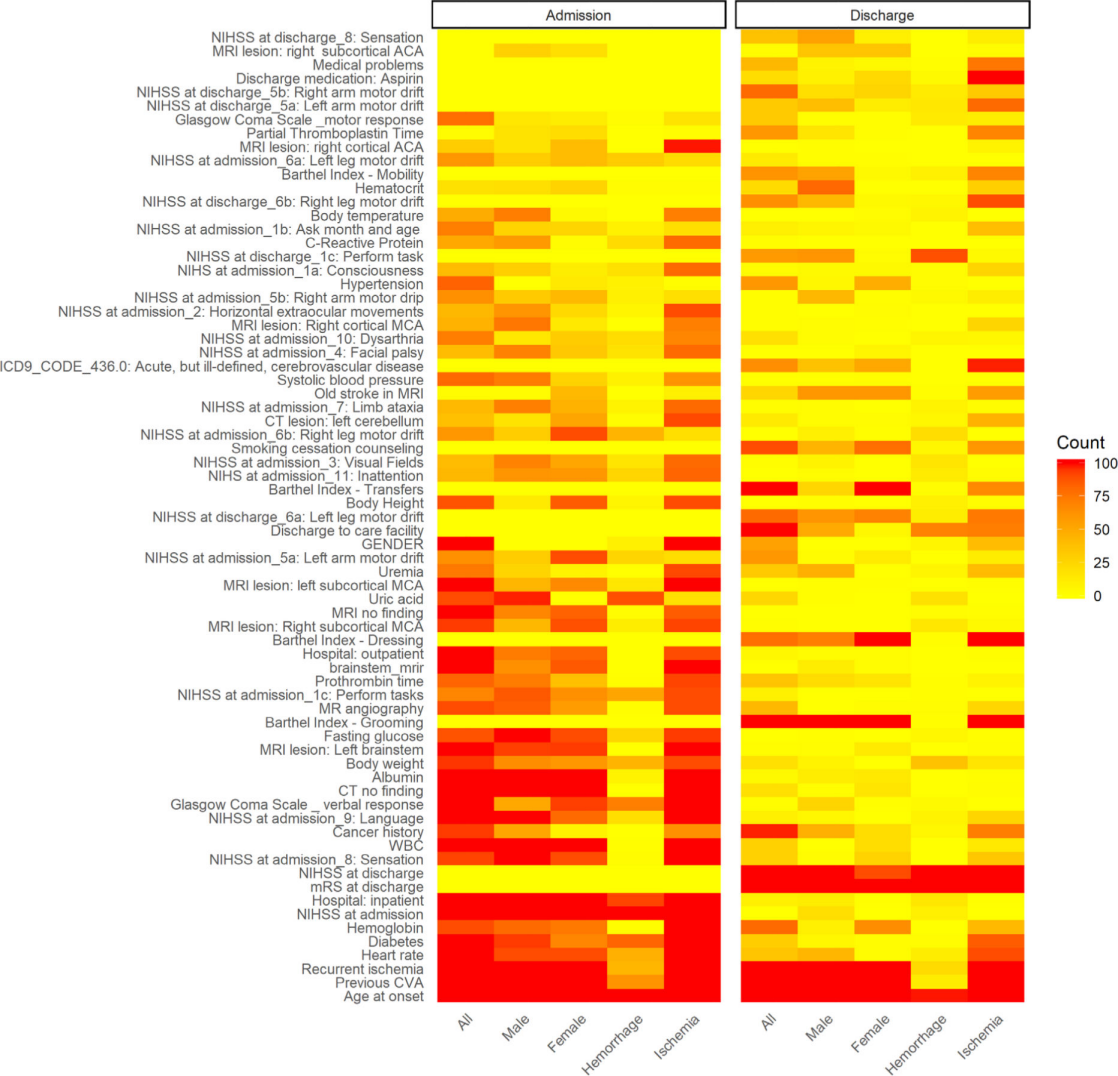
Heatmap of selected variables. The counts of selected variables were calculated from 100/100 times computation. More variables were selected in the admission models indicating more clinical variables were needed to achieve good performance in outcome prediction. Age of onset and previous CVA were selected most frequently in both admission and discharge models among different subgroups. The variables in white color were not included (i.e., not available) in the models assessed. CVA: cerebral vascular accident

Figure 2 shows the clinical variables selected in 100/100 times of computation and selection (see Methods section) at admission and discharge, presented as a heatmap. It was found that more variables were selected in models at admission (left-side columns) comparing with those at discharge (right-side columns), indicating that more variables were needed in at admission models to achieve the desired performance for outcome prediction compared to those at discharge. Also, the variables of NIHSS at admission and the mRS at discharge were found being selected 100/100 times when modeling at each time point (i.e., admission *vs*. discharge), indicating their important roles in predicting patient’s functional outcome at 90-day follow-up. Age of onset and history of previous cerebral vascular accident (CVA) were the most frequently selected variables at both time points among different subgroups of patients. Other most selected clinical variables were found from functional assessments, such as the history of illness and blood tests. It is interesting to note that different numbers of variables were selected in male, female, hemorrhagic, and ischemic patients to achieve desired performance in prediction, which might be related to the different sample sizes in the dataset and characteristics of each patient subgroups.

**Figure 3.**
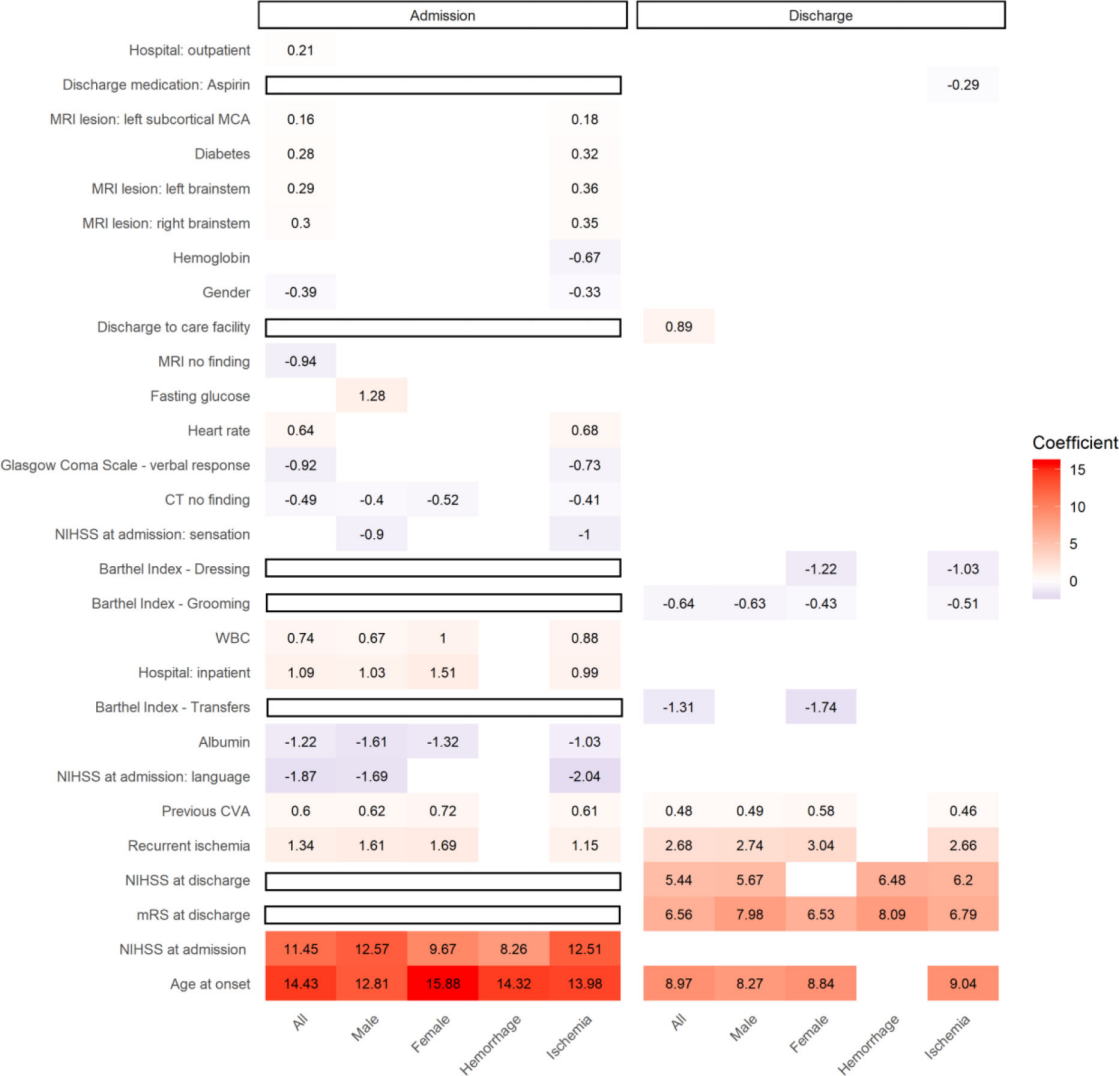
The coefficients of selected clinical variables. The variables shown were selected 100/100 times, and the coefficients were calculated in the LR models. The higher number of the coefficient indicated the degree of importance in predicting the functional outcome; for example, age at onset and functional assessments were higher than those of other clinical variables. In addition, the sign (+ or −) were indicative of positive or negative impacts on the prediction outcomes. The variables in the blank rectangle were not included (i.e., not available) in the model assessed. LR: logistic regression

To further compare and evaluate the potential effects that each variable contributed to the outcome prediction models, the coefficients of 100/100 times selected variables calculated in the LR models are shown in Figure 3. The coefficients of the LR model represented the influence of variables on the prediction target^[23]^. In our study, the coefficients of age at onset and functional assessments, including NIHSS at admission, mRS at discharge, and NIHSS at discharge were found to be higher than those of other clinical variables, indicating their importance in contributing to the prediction models for functional outcomes. Other variables, including medical history (recurrent ischemia, previous CVA, and diabetes), Barthel index (transfer, grooming, and dressing), lesions in CT and MRI, blood tests (albumin, white blood cell count, fasting glucose, and hemoglobin), the origin of hospitalization (from inpatient and outpatient), and discharge medication (aspirin) were also found to be significant contributors to our outcome prediction models. These selected variables may provide insights into understanding the stroke profiles unique to the population studied. The adjusted odds ratio of variables selected in the admission model is shown in Supplemental Figure S2.

## DISCUSSION

Previous clinical studies have shown that age and gender are important factors for stroke outcome prediction^[24–27]^ in different populations. In the present study, the odds ratio of age was found to be only 1.05 in our population, and male gender was found to be predominant (65.0%) in the good outcome group [Table 1], indicating that age and gender differences contributed and correlated to their clinical outcomes but differed in the populations studied. The average onset age of males (65.1 ± 13.2) was younger than that of females (69.3 ± 13.1) in our population, and the difference in ages between males and females has been found to play an important role in their functional outcome^[28]^, which was consistent with our findings. There were also several clinical factors reported, including pathology and the effectiveness of treatment that affected the gender difference of stroke outcome^[29–31]^.

Patients diagnosed with ischemia were found to associate with good outcomes (92.0%) in our population. Even though the average onset age of hemorrhagic patients (60.9 ± 14.7) was found to be younger than that of ischemic patients (67.4 ± 13.0), their NIHSS at admission was found to be higher (more severe) in hemorrhagic patients (8.5 ± 8.8) than that in ischemic patients (5.7 ± 6.5), indicating that hemorrhagic patients in our population were in the more severe condition when admitted. The differences of NIHSS between hemorrhagic and ischemic stroke at admission have been reported in other studies^[32,33]^. In our study, the improvement of NIHSS in hemorrhagic patients (−2.3 ± 6.1) during admission was found to be significantly greater, indicating significantly better recovery than that in ischemic patients (−1.3 ± 4.3). Hematoma expansion, edema formation, and increased intracranial pressure were likely contributors to the outcome^[34,35]^. Even with significant improvement found during triage, the mRS_3m was higher in those hemorrhagic patients (2.4 ± 1.7) than that in ischemic patients (2.0 ± 1.6), indicating that inherent damage occurred in older hemorrhagic patients in our population.

Patients with underlying diseases except for hyperlipidemia (OR = 0.83–0.90) were found to have poor stroke outcomes. It has been reported that hyperlipidemia is related to favorable stroke outcome^[36]^. In the present study, we showed that smoking and drinking were related to good outcomes with OR 0.59 (0.56–0.62) and 0.60 (0.56–0.64) in our population, respectively. The impacts of smoking and drinking on stroke outcomes have been found to be controversial^[37–39]^. Potential confounders should be considered, since smoking and drinking were not selected in our final prediction models [Figure 3]. The relationship between stroke outcomes and hospital distances, socioeconomic status, and timely treatment has been previously discussed in the literature^[40]^. Our study suggested that patients treated at close by medical centers had good outcomes, and so were those in the east part of Taiwan (rural countryside) with farther distance to the hospital, which may be associated with a younger population and smaller sample size, although further studies may be required to explain this effect.

In our study, age of onset and previous CVA were found to be the most frequently selected predictors [Figure 2], which were consistent with previously reported studies, however, these two variables were non-modifiable factors which make them unusable for triage or treatment. Nevertheless, some variables selected in our models might provide useful guidance during the triage and for the treatment plan. For example, according to the coefficients of LR selected variables in different patient subgroups [Figure 3], the models for all patients at admission required fewer variables to achieve similar performance as those of all 140 variables used. In the case of the hemorrhagic patients, only two variables were selected that might occur due to the different natural courses and pathology between hemorrhagic and ischemic patients^[34,35]^. The negative coefficients of albumin and hemoglobin found in our study indicated higher values might improve stroke outcomes. On the contrary, the positive coefficients of white blood cells (WBC), fasting sugar, and heart rate provided warning signs to clinicians that these variables might be prone to poor outcomes. The negative coefficient of Aspirin prescribed as the discharge medication was also shown a positive effect in our discharge model of ischemic patients. The associations between stroke outcomes and albumin^[41]^, hemoglobin^[42]^, and WBC^[43]^ have been reported, but Aspirin prescription has not been shown as beneficial to stroke outcomes^[44]^ as found in our current population study. For the potential optimal options of treatment, further evaluations on Aspirin were required for targeted interventions to prove its positive effort on the improvement of stroke outcomes. Furthermore, several imaging variables as shown in Supplemental Figure S2 including MRI no Finding (OR = 0.37), CT no Finding (OR = 0.62), MRI Lesion: Left subcortical MCA (OR = 1.58), MRI: Left brainstem (OR = 1.85), and MRI Lesion: Right brainstem (OR = 2.03), were selected in our admission prediction models. These clinical imaging findings can be used as early predictors and indicators for predicting stroke outcomes to alert and assist clinicians during triages of stroke patients.

Several studies have tried to build different prognostic models aiming for stroke outcome predictions [Table 3] using various sample sizes in different populations. For example, Counsell *et al.*^[45]^ utilized six variables to predict 30 days of survival with 0.84 to 0.88 AUC. Muscari *et al.*^[46]^ proposed a multiple regression model to predict 9-month mRS with an AUC of 0.84. Teale *et al.*^[8]^ reviewed 17 models using two to eleven variables to predict 30–180 days outcome, and their AUCs ranged from 0.75 to 0.88. Wouters *et al.*^[27]^ built a multivariate model utilizing baseline NIHSS and age to predict 90-day mRS, and the AUC was 0.86. Jampathong *et al.*^[25]^ reviewed 23 prognostic models for complete recovery in ischemic stroke, and the pooled AUC of these models was 0.78. Although different prognostic models were attempted with reasonable performance, they were built to a unique model with fewer cases and specific populations^[8]^. This study proposed unique prognostic models for the nationwide Taiwanese population with significant performance improvements than previously described. In our study, the AUC of our statistical LR models at admission and discharge were 0.85–0.87 and 0.95–0.96 higher than any previously reported and with fewer selected features between 2–18 and 2–8 in four different subgroups (male, remale, ischemia, and hemorrhage). In addition, the sample sizes of previous studies ranged only from hundreds to thousands, and this study employed 37,094 stroke patients with high-quality datasets that were clinically validated by machine learning methods previously reported^[11]^.

Our current study has some limitations that may have prevented us from achieving even greater performance. First, the prediction model was based on a prospective cohort study dataset in a specific population based on TSR; thus, our specific findings were limited to variables available from the registry. Some important prognostic variables were not included, such as pre-stroke medical history, previous acute events, lifestyle information, and socioeconomic status. Second, heparin, IA thrombolysis, IV t-PA, Foley, and rehabilitation showed strong adverse effects on stroke outcomes [Table 1]; the results were likely confounded by indications (e.g., stroke severity), and relatively unbalanced case numbers in each subgroup, and these factors were not selected in the final models. In a future study, we aim to build separate models for specific patient populations to improve performance further and work toward establishing the clinical tools to help improve stroke care and outcomes.

In conclusion, modeling of clinical assessment variables for stroke outcome prediction was found to be population-specific. The study proposed prognostic models for predicting stroke outcomes with exceptional performance that employed a significantly large sample size of nationwide stroke patients of the Taiwanese population. Our study identified important clinical variables collected at admission and discharge to build prediction models in four different patient subgroups, and these variables can be further reduced to only a few (2–18) variables with similar performance. The results might provide insight information for interventions to improve stroke care and outcomes. Our proposed models achieved significantly better prediction performance than previously reported models. It should be noted that prognostication or triage in the acute stroke period is critical but complicated, and current prediction models will need to be further investigated and validated in prospective studies before being developed into useful tools to assist clinicians in emergency settings.

## Supplementary Material

2

## Figures and Tables

**Table 1.** Comparison of clinical data selected between good and poor outcome patients employed in this study

	Good outcome (*N* = 23,586, 63.6%)	Poor outcome (*N* = 13,508, 36.4%)	OR	CI	*P* value
Age, mean (± SD)	63.9 (± 12.8)	71.8 (± 12.6)	1.05	1.05–1.05	< 0.001
Male sex, *n* (%)	15,322 (65.0%)	6972 (51.6%)	0.58	0.55–0.60	< 0.001
BMI, mean (± SD)	24.9 (± 3.5)	23.9 (± 3.8)	0.93	0.92–0.93	< 0.001
Admission days, mean (± SD)	6.2 (± 4.0)	10.3 (± 7.0)	1.16	1.15–1.17	< 0.001
Ischemia, *n* (%)	21,695 (92.0%)	11,816 (87.5%)	0.61	0.57–0.65	< 0.001
Medical history, *n* (%)					
Hypertension	18,097 (76.7%)	11,189 (82.8%)	1.46	1.39–1.55	< 0.001
Diabetes	8522 (36.1%)	5939 (44.0%)	1.39	1.33–1.45	< 0.001
Dyslipidemia	12,446 (52.8%)	6631 (49.1%)	0.86	0.83–0.90	< 0.001
Previous CVA	5123 (21.7%)	5358 (39.7%)	2.37	2.26–2.48	< 0.001
Heart disease	6402 (27.1%)	5238 (38.8%)	1.70	1.63–1.78	< 0.001
Cancer	383 (1.6%)	410 (3.0%)	1.90	1.65–2.18	<0.001
Uremia	415 (1.8%)	437 (3.2%)	1.87	1.63–2.14	< 0.001
Smoking	9760 (41.4%)	3969 (29.4%)	0.59	0.56–0.62	< 0.001
Drinking	3632 (15.4%)	1320 (9.8%)	0.60	0.56–0.64	< 0.001
Area, *n* (%)					
North	9839 (41.7%)	3702 (27.4%)	2.58	2.08–3.21	< 0.001
Middle	7812 (33.1%)	6740 (49.9%)	5.92	4.76–7.36	< 0.001
South	5283 (22.4%)	2971 (22.0%)	3.86	3.10–4.81	< 0.001
East	652 (2.8%)	95 (0.7%)	Reference	Reference	Reference
Hospital Scale, *n* (%)					
Medical Center	14,445 (61.2%)	5389 (39.9%)	0.42	0.40–0.85	< 0.001
Regional Hospital	9141 (38.8%)	8095 (59.9%)	Reference	Reference	Reference
Functional assessment, mean (SD)					
NIHSS at admission	3.34 (± 3.64)	10.57 (± 8.37)	1.26	1.25–1.27	< 0.001
NIHSS at discharge	1.87 (± 2.06)	9.36 (± 7.43)	1.67	1.65–1.68	< 0.001
mRS at discharge	1.51 (± 1.00)	3.96 (± 0.94)	8.38	8.03–8.75	< 0.001
Laboratory data, mean (SD)					
WBC, 10^9/L	7.71 (± 2.18)	8.10 (± 2.37)	1.08	1.07–1.09	< 0.001
Hemoglobin, g/dL	13.98 (± 1.78)	13.35 (± 1.90)	0.83	0.82–0.84	< 0.001
Albumin, g/dL	3.66 (± 0.28)	3.58 (± 0.34)	0.41	0.38–0.44	< 0.001
Fasting glucose, mg/dL	116.04 (± 23.11)	120.50 (± 23.92)	1.01	1.01–1.01	< 0.001
TC, mg/dL	149.44 (± 54.6)	144.06 (± 53.28)	0.99	0.99–0.99	< 0.001
TG, mg/dL	166.23 (± 51.53)	155.27 (± 53.85)	0.99	0.99–0.99	< 0.001
Treatment, *n* (%)					
Aspirin	6093 (25.8%)	3599 (26.6%)	0.46	0.44–0.48	< 0.001
Heparin	505 (2.1%)	464 (3.4%)	1.63	1.43–1.85	< 0.001
IA thrombolysis	229 (1.0%)	452 (3.3%)	3.53	3.01–4.15	< 0.001
IV t-PA	473 (2.0%)	408 (3.0%)	1.52	1.33–1.74	< 0.001
Foley	1553 (6.6%)	4496 (33.3%)	7.08	6.65–7.54	< 0.001
Rehabilitation	10,587 (44.9%)	10,404 (77.0%)	4.12	3.92–4.32	< 0.001

OR: odds ratio; CI: 95% confidence interval; WBC: white blood cells; TC: total cholesterol; TG: triglycerides; IA thrombolysis: intra-arterial thrombolysis; IV t-PA: intravenous tissue plasminogen activator

**Table 2.** Performance of stroke outcome predictions using all and LR-selected clinical data at admission and discharge

Time point	Admission					Discharge				
Model	Feature^#^	Sen	Spe	Accuracy	AUC	Feature^#^	Sen	Spe	Accuracy	AUC
With all Features										
All	140	0.63	0.91	0.81	0.87	262	0.84	0.93	0.90	0.96
Male	140	0.59	0.92	0.82	0.86	262	0.82	0.94	0.90	0.95
Female	140	0.70	0.87	0.79	0.87	262	0.86	0.92	0.90	0.96
Ischemia	140	0.64	0.91	0.82	0.88	262	0.83	0.94	0.90	0.96
Hemorrhage	140	0.75	0.81	0.78	0.85	262	0.87	0.86	0.87	0.92
With LR-Selected Features										
All	18	0.59	0.91	0.79	0.86	8	0.84	0.93	0.90	0.96
Male	11	0.55	0.92	0.81	0.85	6	0.82	0.93	0.90	0.95
Female	8	0.67	0.87	0.78	0.85	7	0.87	0.92	0.90	0.96
Ischemia	18	0.61	0.91	0.81	0.87	8	0.83	0.93	0.90	0.96
Hemorrhage	2	0.72	0.84	0.79	0.85	2	0.85	0.89	0.87	0.95

# Number of variables selected; Sen: sensitivity; Spe: specificity

**Table 3.** Comparison of variables selected by different prognostic models in the literature

Author (year)	Sample size	Outcome assessed	Variables included in the model	Performance (AUC)
Counsell *et al.*^[45]^ (2002)	530	30-day mortality and six-month independent survival	age, living alone, independence before stroke, verbal component of GCS, arm strength, ability to walk	0.84–0.88
Muscari *et al.*^[46]^ (2011)	211	9-month mRS	NIHSS, need of urinary catheter, oxygen administration, upper limb paralysis	0.84
Teale *et al.*^[8]^ (2012) review 17 models	27–8964	30–180 days functional assessment	2–11 variables (age, NIHSS, limb weakness, dysarthria, conscious, diabetes, previous stroke, fever, mRS, *etc*.)	0.75–0.88
Wouters *et al.*^[27]^ (2018)	369	90 days mRS	Baseline-NIHSS, age, ischemic heart disease	0.86
Jampathong *et al.*^[25]^ (2018) review 23 models	75–4441	90–365 days functional assessment	1–11 variables (NIHSS, age, infarct volume, diabetes, previous stroke, pre-stroke disability, small-vessel stroke, t-PA use, preadmission mRS, sex, atrial fibrillation,..,etc.)	0.73–0.84
Proposed model by LR method (This study)	37,094	90-day mRS	age, discharge mRS, discharge NIHSS, recurrent ischemia, previous stroke, Barthel index (BI)-grooming, BI-dressing, aspirin use	0.95–0.96

NIHSS: National Institutes of Health Stroke Scale; AUCs: area under the curves
