# Supplementary material for: Comparison of outcome prediction models post-stroke for a population-based registry with clinical variables collected at admission vs. discharge
Source: Vessel Plus. Author manuscript; Available in PMC 2022 Mar 29. (PMC8963213)
Supplement: 2 [file NIHMS1739597-supplement-2.pdf]

Comparison of Outcome Prediction Models Post Stroke for a Population-specific Registry with Clinical Variables Collected at Admission vs. Discharge

Running Title: Population-specific Stroke Outcome Prediction

Kai-Cheng Hsu [1,2,3], Ching-Heng Lin [4], Kory R. Johnson[5], Yang C. Fann[5*], Chung Y. Hsu[6], Chon-Haw Tsai[3], Po-Lin Chen[7], Wei-Lun Chang[8], Po-Yen Yeh[9], Cheng-Yu Wei[10], and Taiwan Stroke Registry Investigators[#]

[1]Artificial Intelligence Center for Medical Diagnosis, China Medical University, Taichung, Taiwan

[2]School of Medicine, College of Medicine, China Medical University, Taichung, Taiwan

[3]Department of Neurology, China Medical University Hospital, Taichung, Taiwan

[4]Center for Artificial Intelligence in Medicine, Chang Gung Memorial Hospital, Taoyuan, Taiwan

[5]Bioinformatics Section, National Institute of Neurological Disorder and Stroke, National Institutes of Health, Bethesda, Maryland, United States

[6]Graduate Institute of Biomedical Sciences, China Medical University, Taichung, Taiwan

[7]Neurological Institute, Taichung Veterans General Hospital, Taichung, Taiwan

[8]Department of Neurology, Show Chwan Memorial Hospital, Changhua County, Taiwan

[9]Department of Neurology, St. Martin De Porres Hospital, Chiayi, Taiwan

[10]Department of Neurology, Chang Bing Show Chwan Memorial Hospital, Changhua County, Taiwan

[#]Listed in supplemental appendix I

Words Count: 3161

Corresponding Author:

Yang C. Fann, Ph.D.

Director, Intramural IT & Bioinformatics Program

National Institute of Neurological Disorders and Stroke

National Institutes of Health

9000 Rockville Pike, Bethesda, Maryland 20892

Tel: 301-451-5153, Fax: 301-480-3563,

E-mail: fann@ninds.nih.gov

Supplemental Data

Figure S1. Flowchart of model construction with different subgroup datasets. All patients datasets were divided into female, male, hemorrhage, and ischemia subgroups. We used 70% data for training and 30% for testing for each subgroup dataset. For two different time points, we used 140 variables for admission and 262 variables for discharge. Variables selected 100/100 times were used in the LR model to compare with another LR model using all variables.

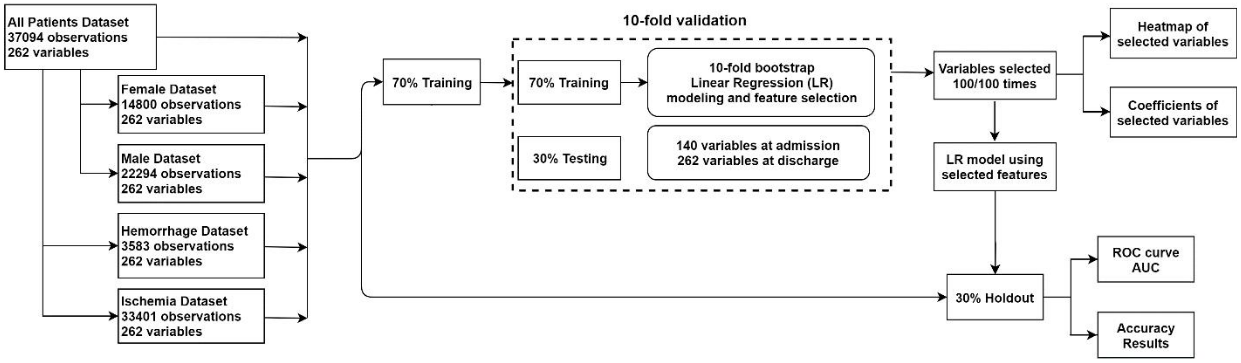

Figure S2. The adjusted odds ratio of selected variables at admission. The odds ratios of no finding of MRI and CT, albumin, NIHSS of language, gender, and verbal response of Glasgow coma scale were less than 1.

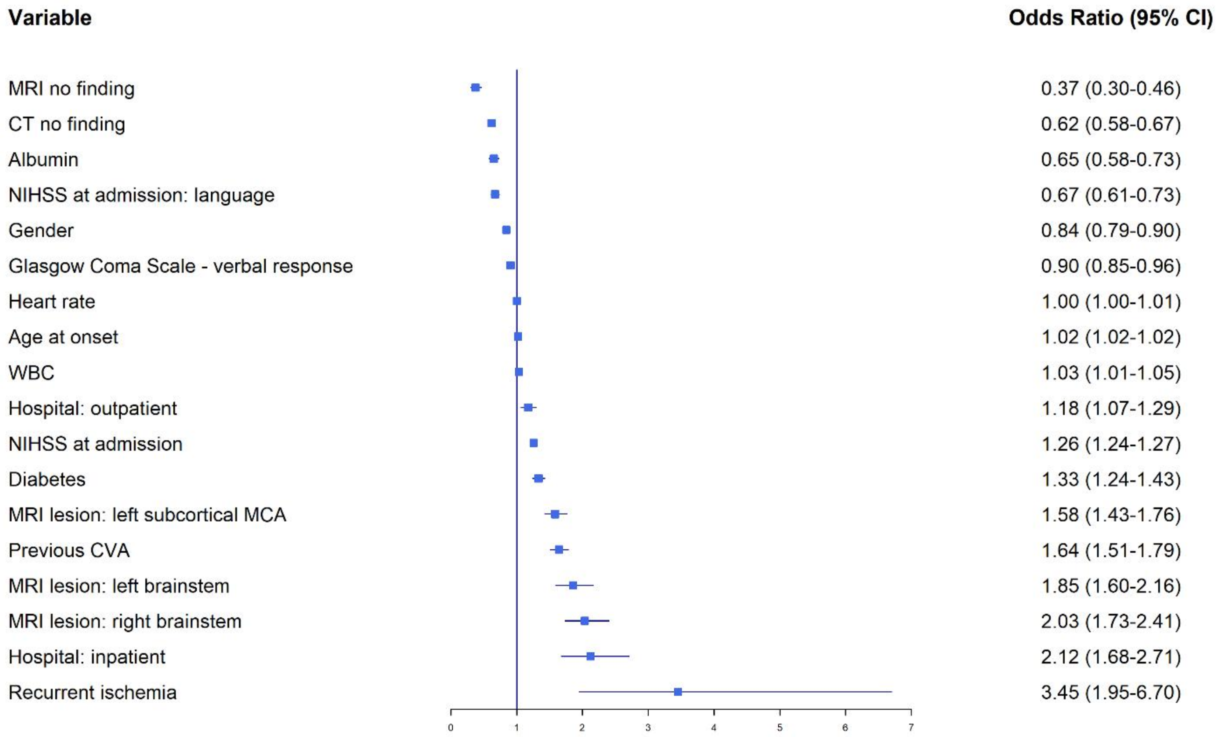

Table S1. Differences of NIHSS and mRS between key time points (admission vs. discharge and discharge vs. 3-month follow-up)

NIHSS_diff			mRS_diff		
Difference	Case	Percentage	Difference	Case	Percentage
**-21 to -30**	**54**	**0.15%**	-5	12	0.03%
**-11 to -20**	**176**	**0.47%**	-4	47	0.13%
**-5 to -10**	**1216**	**3.28%**	-3	392	1.06%
**-4**	**5204**	**14.03%**	-2	2388	6.44%
-3	2877	7.76%	-1	10605	28.59%
-2	5059	13.64%	0	21310	57.45%
-1	5973	16.10%	1	1687	4.55%
0	10659	28.74%	2	409	1.10%
1	1798	4.85%	3	146	0.39%
2	1192	3.21%	4	76	0.20%
3	809	2.18%	5	20	0.05%
4	514	1.39%	6	2	0.01%
5 to 10	1068	2.88%			
11 to 20	392	1.06%			
21 to 30	94	0.25%			
31 to 40	9	0.02%			

NIHSS_diff: NIHSS_out – NIHSS_in; mRS_diff: mRS_3m – mRS_out;

Bold number: significant improvement

Appendix I: List of Taiwan Stroke Registry Investigators:

China Medical University Hospital: Yuh-Cherng Guo (Principal Investigator), Chon-Haw Tsai, Wei-Shih Huang, Chung-Ta Lu, Tzung-Chang Tsai, Chun-Hung Tseng, Kang-Hsu Lin, Woei-Cherng Shyn, Yu-Wan Yang, Yen-Liang Liu, Der-Yang Cho, Chun-Chung Chen, Chung-Hsiang Liu
National Taiwan University Hospital: Jiann-Shing Jeng (Principal Investigator), Sung-Chun Tang, Li-Kai Tsai, Shin-Joe Yeh
E-Da Hospital / I-Shou University: Shih-Pin Hsu (Principal Investigator), Han-Jung Chen, Cheng-Sen Chang, Hung-Chang Kuo, Lian-Hui Lee, Huan-Wen Tsui, Jung-Chi Tsou, Yan-Tang Wang, Yi-Cheng Tai,Kun-Chang Tsai, Yen-Wen Chen, Kan Lu, Po-Chao Liliang, Yu-Tun Tsai, Cheng-Loong Liang, Kuo-Wei Wang, Hao-Kuang Wang, Jui-Sheng Chen, Po-Yuan Chen, Cien-Leong Chye, Wei-Jie Tzeng, Pei-Hua Wu
National Cheng Kung University Hospital: Chih-Hung Chen (Principal Investigator), Pi-Shan Sung, Han-Chieh Hsieh, Hui-Chen Su
Shin Kong WHS Memorial Hospital: Hou-Chang Chiu (Principal Investigator), Li-Ming Lien, Wei-Hung Chen, Chyi-Huey Bai, Tzu-Hsuan Huang, Chi-Ieong Lau,Ya-Ying Wu, Hsu-Ling Yeh, Anna Chang
Kaohsiung Veterans General Hospital: Ching-Huang Lin (Principal Investigator), Cheng-Chang Yen
Kaohsiung Medical University Chung-Ho Memorial Hospital: Ruey-Tay Lin (Principal Investigator), Chun-Hung Chen, Gim-Thean Khor, A-Ching Chao, Hsiu-Fen Lin, Poyin Huang
Chi Mei Medical Center: Huey-Juan Lin (Principal Investigator), Der-Shin Ke, Chia-Yu Chang, Poh-Shiow Yeh, Kao-Chang Lin, Tain-Junn Cheng, Chih-Ho Chou, Chun-Ming Yang, Hsiu-Chu Shen
Chung Shan Medical University Hospital: An-Chih Chen (Principal Investigator), Shih-Jei Tsai, Tsong-Ming Lu, Sheng-Ling Kung, Mei-Ju Lee, Hsi-Hsien Chou
Show Chwan Memorial Hospital: Hsin-Yi Chi (Principal Investigator), Chou-Hsiung Pan, Po-Chi Chan, Min-Hsien Hsu, Wei-Lun Chang,Ya-Ying Wu , Zhi-Zang Huang , Hai-Ming Shoung,Yi-Chen Lo, Fu-Hwa Wang
Cheng Hsin General Hospital: Ta-Chang Lai (Principal Investigator), Jiu-Haw Yin, Chung-JenWang, Kai-ChenWang, Li-Mei Chen, Jong-Chyou Denq
En Chu Kong Hospital: Yu Sun (Principal Investigator), Chien-Jung Lu, Cheng-Huai Lin, Chieh-Cheng Huang, Chang-Hsiu Liu, Hoi-Fong Chan
Far Eastern Memorial Hospital: Siu-Pak Lee (Principal Investigator)
Kuang Tien General Hospital: Ming-Hui Sun (Principal Investigator), Li-Ying Ke
Taichung Veterans General Hospital: Po-Lin Chen (Principal Investigator), Yu-Shan Lee
Ditmanson Medical Foundation Chia-Yi Christian Hospital: Sheng-Feng Sung(Principal Investigator), Cheung-Ter Ong, Chi-Shun Wu, Yung-Chu Hsu, Yu-Hsiang Su, Ling-Chien Hung
Tri-Service General Hospital: Jiunn-Tay Lee (Principal Investigator), Jiann-Chyun Lin, Yaw-Don Hsu, Jong-Chyou Denq, Giia-Sheun Peng, Chang-Hung Hsu, Chun-Chieh Lin, Che-Hung Yen, Chun-An Cheng, Yueh-Feng Sung, Yuan-Liang Chen, Ming-Tung Lien, Chung-

Hsing Chou, Chia-Chen Liu, Fu-Chi Yang, Yi-ChungWu, An-Chen Tso, Yu- Hua Lai, Chun-I Chiang, Chia-Kuang Tsai, Meng-Ta Liu, Ying-Che Lin, Yu-Chuan Hsu

Cathay General Hospital: Tsuey-Ru Chiang (Principal Investigator), Mei-Ching Lee, Pai-Hao Huang, Sian-King Lie, Pin-Wen Liao, Jen-Tse Chen

Changhua Christian Hospital: Mu-Chien Sun (Principal Investigator), Tien-Pao Lai, Wei-Liang Chen, Yen-Chun Chen, Ta-Cheng Chen, Wen-Fu Wang, Kwo-Whei Lee, Chen-Shu Chang, Chien-Hsu Lai, Siao-Ya Shih, Chieh-Sen Chuang, Yen-Yu Chen, Chien-Min Chen

Taipei Tzuchi Hospital: Shinn-Kuang Lin (Principal Investigator, School of Medicine, Tzuchi University, Hualien, Taiwan), Yu-Chin Su, Cheng-Lun Hsiao, Fu-Yi Yang, Chih-Yang Liu, Han-Lin Chiang.

Min Sheng General Hospital: Chun-Yuan Chang (Principal Investigator), I-sheng Lin,Chung-Hsien Chien,Yang-Chuang Chang

Lin Shin Hospital: Chih-Hao Lin (Principal Investigator), Pai-Yi Chiu

National Taiwan University Hospital Yunlin Branch: Yu-Jen Hsiao (Principal Investigator), Chen-Wen Fang

Landseed Hospital: Yu-Wei Chen (Principal Investigator), Kuo-Ying Lee, Yun-Yu Lin, Chen-Hua Li, Hui-Fen Tsai, Chuan-Fa Hsieh, Chih-Dong Yang, Shiumn-Jen Liaw, How-Chin Liao

Cheng Ching General Hospital: Shoou-Jeng Yeh (Principal Investigator), Ling-Li Wu, Liang-Po Hsieh, Yong-Hui Lee, Chung-Wen Chen

China Medical University Beigang Hospital: Chih-Shan Hsu(Principal Investigator),Ye-Jian-Jhih, Hao-Yu Zhuang, Yan-Hong Pan, Shin-An Shih

Taipei Medical University -Wan Fang Hospital: Chin-I Chen (Principal Investigator), Jia-Ying Sung, , Hsing-Yu Weng, Hao-Wen Teng, Jing-Er Lee, Chih-Shan Huang, Shu-Ping Chao

Taipei Medical University Hospital: Rey-Yue Yuan (Principal Investigator), , Jau- Jiuan Sheu, Jia-Ming Yu, Chun-Sum Ho, Ting-Chun Lin

Kuang Tien General Hospital Dajia Division: Shih-Chieh Yu(Principal Investigator)

Changhua Christian Hospital Yunlin Branch: Jiunn-Rong Chen (Principal Investigator), Song-Yen Tsai

Chang Bing Show Chwan Memorial Hospital: Cheng-Yu Wei (Principal Investigator), Tzu-Hsuan Huang, Chao-Nan Yang, Chao-Hsien Hung, Ian Shih

Lotung Poh Ai Hospital: Hung-Pin Tseng (Principal Investigator), Chin-Hsiung Liu, Chun-Liang Lin, Hung-Chih Lin, Pi-Tzu Chen

Taipei Medical University - Shuang Ho Hospital: Chaur-Jong Hu (Principal Investigator), Nai-Fang Chi, Lung Chan

Taipei Veterans General Hospital & National Yang-Ming University School of Medicine: Chang-Ming Chern (Principal Investigator), Chun-Jen Lin, Shuu-Jiun Wang, Li-Chi Hsu, Wen-Jang Wong, I-Hui Lee, Der-Jen Yen, Ching-Piao Tsai, Shang-Yeong Kwan, Bing-Wen Soong, Shih-Pin Chen, Kwong-Kum Liao, Kung-Ping Lin, Chien Chen, Din-E Shan, Jong-Ling Fuh, Pei-Ning Wang, Yi-Chung Lee, Yu-Hsiang Yu, Hui-Chi Huang, Jui-Yao Tsai

Chi Mei Medical Center, Liouying: Ming-Hsiu Wu (Principal Investigator), Shi-Cheng Chen, Szu-Yi Chiang, Chiung-Yao Wang

Buddhist Dalin Tzu Chi General Hospital: Ming-Chin Hsu (Principal Investigator)

St. MARTIN DE PORRES HOSPITAL: Chien-Chung Chen (Principal Investigator), Po-Yen Yeh, Yu-Tai Tsai, Ko-Yi Wang

Sin-Lau Hospital, Tainan, the Presbyterian Church in Taiwan: Tsang-Shan Chen(Principal Investigator)
Cardinal Tien Hospital: Ping-Keung Yip (Principal Investigator), Vinchi Wang, Kaw-ChenWang, Chung-Fen Tsai, Chao-Ching Chen, Chih-Hao Chen, Yi-Chien Liu, Shao-Yuan Chen, Zi-Hao Zhao, Zhi-Peng Wei
Yumin Medical Corporation Yumin Hospital: Shey-Lin Wu(Principal Investigator)
Kaohsiung Municipal Hsiao-kang Hospital: Ching-Kuan Liu(Principal Investigator)
Wei Gong Memorial Hospital: Ryh-Huei Lin (Principal Investigator), Ching-Hua Chu
Taipei City Hospital Ren Ai Branch: Sui-Hing Yan (Principal Investigator), Yi-Chun Lin, Pei-Yun Chen, Sheng-Huang Hsiao
National Taiwan University Hospital Hsin-Chu Branch: Bak-Sau Yip (Principal Investigator), Pei-Chun Tsai,Ping-Chen Chou, Tsam-Ming Kuo, Yi-Chen Lee, Yi-Pin Chiu, Kun-Chang Tsai
Taichung Hospital Department of Health : Yi-Sheng Liao (Principal Investigator)
Tainan Municipal An-Nan Hospital-China Medical University: Ming-Jun Tsai (Principal Investigator), Hsin-Yi Kao
